# xMDFF: molecular dynamics flexible fitting of low-resolution X-ray structures

**DOI:** 10.1107/S1399004714013856

**Published:** 2014-08-29

**Authors:** Ryan McGreevy, Abhishek Singharoy, Qufei Li, Jingfen Zhang, Dong Xu, Eduardo Perozo, Klaus Schulten

**Affiliations:** a Beckman Institute for Advanced Science and Technology, University of Illinois at Urbana-Champaign, Urbana, IL 61801, USA; b Department of Biochemistry and Molecular Biology, The University of Chicago, Chicago, IL 60637, USA; c Department of Computer Science, University of Missouri, Columbia, MO 65211, USA; d Department of Physics, University of Illinois at Urbana-Champaign, Urbana, IL 61801, USA

**Keywords:** xMDFF, molecular dynamics flexible fitting

## Abstract

X-ray crystallography remains the most dominant method for solving atomic structures. However, for relatively large systems, the availability of only medium-to-low-resolution diffraction data often limits the determination of all-atom details. A new molecular dynamics flexible fitting (MDFF)-based approach, xMDFF, for determining structures from such low-resolution crystallographic data is reported. xMDFF employs a real-space refinement scheme that flexibly fits atomic models into an iteratively updating electron-density map. It addresses significant large-scale deformations of the initial model to fit the low-resolution density, as tested with synthetic low-resolution maps of d-ribose-binding protein. xMDFF has been successfully applied to re-refine six low-resolution protein structures of varying sizes that had already been submitted to the Protein Data Bank. Finally, *via* systematic refinement of a series of data from 3.6 to 7 Å resolution, xMDFF refinements together with electro­physiology experiments were used to validate the first all-atom structure of the voltage-sensing protein Ci-VSP.

## Abstract 2

A new real-space refinement method for low-resolution X-ray crystallography is presented. The method is based on the molecular dynamics flexible fitting protocol targeted at addressing large-scale deformations of the search model to achieve refinement with minimal manual intervention. An explanation of the method is provided, augmented by results from the refinement of both synthetic and experimental low-resolution data, including an independent electrophysiological verification of the xMDFF-refined crystal structure of a voltage-sensor protein.

## 1. Introduction   

X-ray crystallography is arguably the most versatile and dominant technique for delivering atomic structures of biomolecules. An increasing number of structures are submitted each year to the Protein Data Bank, with over 90% of the current entries coming from X-ray crystal structures. Traditional methods for determining X-ray structures include least-squares with gradient descent (Hendrickson, 1985 ▶), maximum likelihood (Pannu & Read, 1996 ▶; Bricogne & Irwin, 1996 ▶; Murshudov *et al.*, 1997 ▶), simulated annealing (Brünger, 1988 ▶) and knowledge-based conformational sampling (Depristo *et al.*, 2005 ▶). However, investigating the structure of large biomolecular complexes has posed a serious challenge to traditional crystallographic techniques. The inherent flexibility of such large systems and the presence of disordered solvent and lipids or ligands often cause the crystals to diffract at low resolutions. Furthermore, in the low-resolution limit the number of atomic coordinates to be determined often exceeds the number of observed diffraction intensities. At moderate to low resolutions, knowledge of the stereochemistry of the system must be incorporated to achieve accurate atomic positions. Lower resolutions, >5 Å, pose a greater challenge to refinement; however, even at ∼7 Å resolution there are in principle enough independent Bragg reflections to determine the backbone torsion angles of protein crystal structures (Brunger *et al.*, 2012 ▶).

Solving structures from low-resolution diffraction data is a difficult, time-consuming process. Consequently, low-resolution data sets are usually chosen to be discarded (Karmali *et al.*, 2009 ▶).

However, new methods are being developed to better handle low-resolution data. For example, DEN refinement incorporates deformable elastic network models with generic stereochemistry and homology information (Schröder *et al.*, 2010 ▶) to address the issue. Other notable recent developments applicable to refining low-resolution structures include normal-mode refinement (Delarue, 2008 ▶), the *Rosetta* implementation of physical energy functions (DiMaio *et al.*, 2011 ▶) and its combination with reciprocal-space X-ray refinement in *PHENIX* (DiMaio *et al.*, 2013 ▶), torsional optimization protocols (Haddadian *et al.*, 2011 ▶) and external structure restraints or jelly-body refinement in *REFMAC* (Murshudov *et al.*, 2011 ▶). Indeed, the number of low-resolution X-ray structures has grown rapidly in recent years (Karmali *et al.*, 2009 ▶).

Here, we present a new method, xMDFF (molecular dynamics flexible fitting for X-ray crystallography), for real-space (Diamond, 1971 ▶; Chapman, 1995 ▶; Chapman & Blanc, 1997 ▶) X-ray refinement. Our method specifically targets the handling of low-resolution data and the large-scale deformations that often separate the target and reference models. To create this method, we extended a previous hybrid method, molecular dynamics flexible fitting (MDFF), developed to solve atomic models from cryo-EM densities. In MDFF, an initial atomic model is subject to a molecular dynamics (MD) simulation with a modified potential energy function that includes a term derived from the cryo-EM density map (Trabuco *et al.*, 2008 ▶, 2009 ▶). The accuracy and robustness of MDFF has been widely demonstrated in many applications solving structural models for the ribosome (Villa *et al.*, 2009 ▶; Gumbart *et al.*, 2009 ▶, 2011 ▶; Becker *et al.*, 2009 ▶; Seidelt *et al.*, 2009 ▶; Trabuco *et al.*, 2010 ▶; Frauenfeld *et al.*, 2011 ▶; Agirrezabala *et al.*, 2011 ▶; Li *et al.*, 2011 ▶), photosynthetic proteins (Hsin *et al.*, 2009 ▶; Sener *et al.*, 2009 ▶) and the first all-atom structure of the HIV capsid (Zhao *et al.*, 2013 ▶). In xMDFF, the MDFF protocol is modified to use iteratively updated model-phased maps to fit and subsequently refine densities derived from low-resolution X-ray diffraction data.

**Figure 1:**
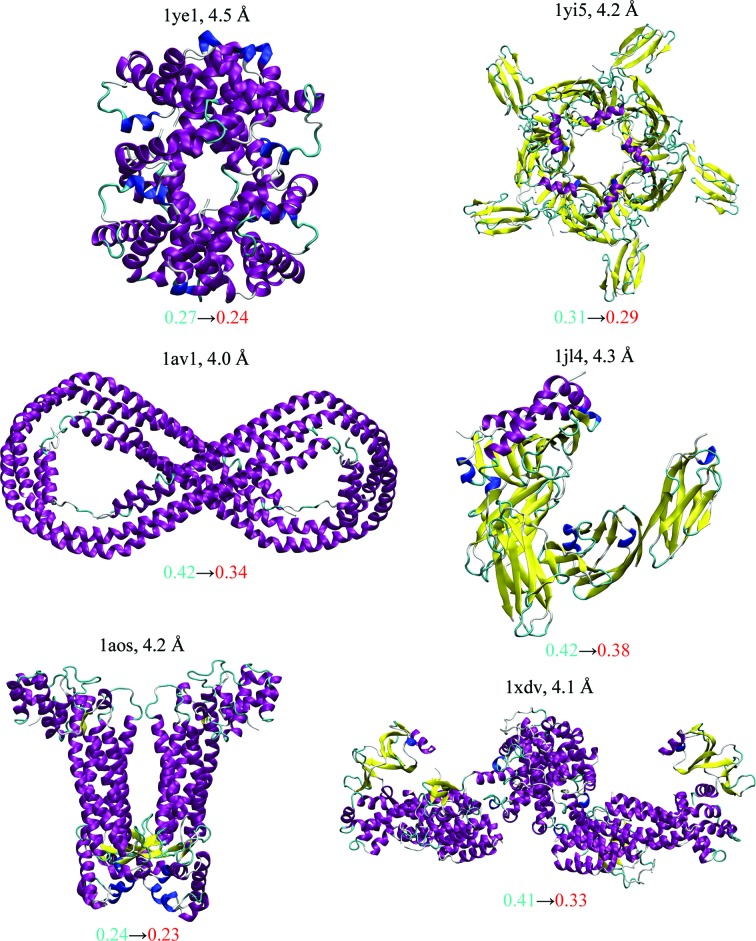
xMDFF re-refinement characteristics for six structures. The structures with diffraction data between 4 and 4.5 Å resolution had been deposited earlier in the Protein Data Bank and were used as initial models for xMDFF refinement. Improvements were evaluated using the *R*_free_ values of the xMDFF-refined structures (shown in red) against their respective starting values taken from the deposited structure (blue). In addition, the increase in the percentage of favored Ramachandran angles owing to xMDFF refinement and other structural statistics, as summarized by the overall *MolProbity* score, were used to measure the improvement in structural geometries. Further details of the refined structures are provided in Table 1 ▶.

xMDFF was tested *via* the refinement of a model with a known final structure at resolutions of 3.5–5 Å. Next, xMDFF successfully further refined six low-resolution (4–4.5 Å) protein structures of varying sizes that had already been submitted to the PDB (Fig. 1 ▶). Finally, xMDFF was applied in parallel with an independent experimental investigation to resolve crystallographic uncertainty in the three-dimensional structure of *Ciona intestinalis* voltage-sensing protein (Ci-VSP). xMDFF refinements were evaluated by (i) *R*_free_, (ii) r.m.s.d. to known targets (test case and voltage-sensing protein) and (iii) improvements in structural geometry of the model. In all cases xMDFF successfully refined the structures and demonstrated an ability to work at very low resolution (7 Å) and with starting models that are very divergent from the target (>5 Å).

## 2. Methods   

### 2.1. Concept   

xMDFF is derived from the MDFF method which solves atomic models of biomolecules imaged by cryo-electron microscopy. In MDFF, an initial atomic model is subjected to an MD simulation with a modified potential energy function that includes a term derived from the cryo-EM density map (Trabuco *et al.*, 2008 ▶, 2009 ▶). Through the density-dependent term, atoms experience steering forces, *f* ^fit^, that locally drive them towards high-density regions, thereby fitting the atoms to the map. For use in low-resolution X-ray crystallography, the MDFF protocol was modified to work with model-phased densities, using the phases ϕ_calc_ calculated from a tentative model and the amplitudes *F*_obs_ from the X-ray diffraction data to produce a 2*mF*_obs_ − *DF*_calc_ density map. The density is biased by the model, but contains sufficient information from the *F*_obs_ to determine the experimental structure. Next, the tentative model is flexibly fitted into the electron-density map using MDFF. In addition to the steering forces derived from the density data, structural restraints are applied to preserve the secondary structure of proteins and nucleic acids (Trabuco *et al.*, 2008 ▶, 2009 ▶), as well as to ensure stereochemical correctness (Schreiner *et al.*, 2011 ▶), thus avoiding overfitting the model into the map. The xMDFF-fitted structure provides new ϕ_calc_ that, together with *F*_obs_, are used to regenerate the electron-density map. The fitted structure is then employed as an updated search model to be driven into the new model-phased density map, and this process continues iteratively. In effect, *f* ^fit^ drives the structure in a direction biased by the *F*_obs_ contribution to the density. Consequently, ϕ_calc_ improvement is indicated by a decrease in *R* factors with each subsequent iteration. The iterations continue until the *R*_free_ and *R*_work_ values reach a minimum or become lower than a predefined tolerance. The quality of the xMDFF-refined all-atom structures is further analyzed *via* computing correlation coefficients (CCs) between the electron-density map generated from the refined ϕ_calc_ with *F*_obs_ and a simulated map at the target resolution.

Next, the MDFF method is briefly discussed in addition to the algorithmic and computational extensions required to address the crystallographic aspects of xMDFF. All of the software required to use xMDFF is currently distributed in released versions of *NAMD* (Phillips *et al.*, 2005 ▶), *VMD* (Humphrey *et al.*, 1996 ▶) and *PHENIX* (Adams *et al.*, 2010 ▶). A script for running xMDFF can be found as part of the Supporting Information^1^ for this article.

### 2.2. MDFF   

xMDFF is a real-space refinement technique and relies on a previously developed method, molecular dynamics flexible fitting (MDFF), which uses a modified potential energy function, *U*_total_, during MD to fit a structure into a low-resolution cryo-EM density (Trabuco *et al.*, 2008 ▶, 2009 ▶). This function has three terms, namely 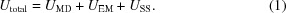
The first term, *U*_MD_, is the conventional MD potential energy function. The second term, *U*_EM_, is a potential energy function derived from the electron density that is used to drive the structure from areas of low density to areas of high density. The third term, *U*_SS_, is a potential which helps to preserve the secondary structure of proteins and nucleic acids through restraints (Trabuco *et al.*, 2008 ▶, 2009 ▶) in addition to ensuring stereochemical correctness in chirality and peptide-bond conformations (Schreiner *et al.*, 2011 ▶). Symmetry restraints can also be introduced if the system exhibits noncrystallographic symmetry (Chan *et al.*, 2011 ▶).

The *VMD* (Humphrey *et al.*, 1996 ▶) plugin *mdff* can be employed to generate a potential energy function *U*_EM_ defined on a three-dimensional grid based on the cryo-EM density map, 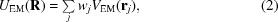
where
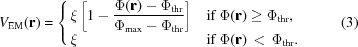
Here Φ(**r**) is the density at position **r**; it and its maximum value Φ_max_ are obtained from the cryo-EM data. In equation (3), a threshold Φ_thr_ is introduced to clamp the Φ(**r**) values that are lower than Φ_thr_ to the Φ_thr_ value, effectively removing the solvent contribution from the map and creating a flat potential for those regions. The global scaling factor ξ uniformly adjusts the strength of the influence of the cryo-EM map on the molecular system. In addition, *V*_EM_(**r**) has a weight *w_j_* for each atom *j* present at position **r** *_j_*. Generally, *w_j_* is set to the atomic mass; this weighting avoids strong differences in the acceleration of atoms owing to mass disparities, ensuring stability of the simulation. This choice of weighting factor is also in line with the rough correspondence between the mass of atoms and their density in a cryo-EM map.

The potential energy function *U*_EM_ defined by (2) and (3) is incorporated into an MD simulation using the *gridForces* feature (Wells *et al.*, 2007 ▶) of *NAMD* (Phillips *et al.*, 2005 ▶). The *gridForces* feature allows an arbitrary potential defined on a three-dimensional grid to be added to an MD simulation. The gradient of the potential is calculated by finite-difference methods and forces are applied to each atom depending on its position on the grid using an interpolation scheme. In the case of MDFF, for each atom *i* in the system, the resulting force is given by
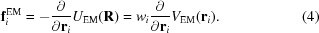
The force **f**_*i*_^EM^ can be tuned *via* the scaling factor ξ (3), which is the same for all atoms, and the weight *w_i_*, which can be defined on a per-atom basis.

### 2.3. xMDFF   

**Figure 2:**
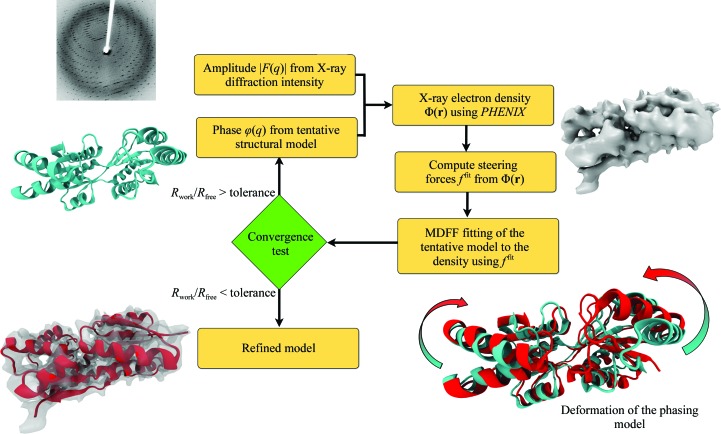
Overview of the xMDFF workflow. Amplitudes *F*_obs_ from the X-ray diffraction data are combined with computed phases ϕ_calc_ from a tentative model to produce an electron-density map Φ(**r**). Biasing forces *f* ^fit^ derived from a Φ(**r**)-dependent potential are used to flexibly fit the phasing model into the map, yielding structures most consistent with the electron density. With the most recently fitted structure as a new phasing model, density maps are synthesized and flexibly fitted until a structure is found with sufficiently low *R*_free_, providing the best possible interpretation of the diffraction data.

To extend MDFF to low-resolution X-ray crystallography and create xMDFF, the MDFF protocol was adjusted to work with electron densities produced from X-ray diffraction instead of a cryo-EM source (Fig. 2 ▶). To this end, xMDFF uses model-phased maps which incorporate the phases ϕ_calc_ from a tentative model and the amplitudes *F*_obs_ from the X-ray diffraction data. Ideally, this approach produces a density, which although biased by the phasing model, is expected to contain a sufficient contribution from the diffraction data such that the density can be used as a target for refinement. However, this may not be the case if the experimental data are too low resolution (>∼7 Å as seen in the Ci-VSP case below) or excessively noisy, or if the phasing model greatly differs from the experimental structure. The model-phased density can be used as a potential (2) to steer the structure into the appropriate locations using MDFF forces (4), now termed *f* ^fit^. Once the structure is fitted into the density, it provides new phases ϕ_calc_ to be used with the *F*_obs_ to generate an updated density. This structure is then fitted into the new map using MDFF, and the process proceeds iteratively until a sufficiently low *R*_free_ is obtained.

To create the densities, xMDFF utilizes tools in the *PHENIX* software suite (Adams *et al.*, 2010 ▶) to generate 2*mF*_obs_ − *DF*_calc_ maps. These maps highlight the areas of the density where the difference between *F*_obs_ and *F*_calc_ is greatest, suggesting that these parts of the structure require refinement. xMDFF employs additional features of *PHENIX* to improve the densities, such as bulk-solvent correction and β-factor sharpening, which improves the maps, particularly at low resolutions (DeLaBarre & Brunger, 2003 ▶). All density maps generated for use in xMDFF exclude the *R*_free_ reflections. This results in poorer quality maps, but allows proper use of the *R*_free_ metric for an unbiased evaluation, which is especially useful for low-resolution data. To help correct for model bias caused by using a homology model to supply phase information, xMDFF employs kicked maps (Pražnikar *et al.*, 2009 ▶), which are produced by randomly perturbing the structure multiple times, calculating densities with the new ϕ_calc_ and averaging the results. Additionally, xMDFF can make use of inherent sampling owing to the MD-based nature of the method and can increase or lower the temperature to control the thermal fluctuations of the system. Conventional MD simulations generate ensembles of atomic structures under constraints such as constant pressure (*P*), volume (*V*), temperature (*T*) and number of particles (*N*). Generally, xMDFF is compatible with any such ensemble-generation scheme, *e.g.* constant *NPT* or *NVT*, and microenvironmental conditions, such as vacuum, explicit/implicit solvent or membrane, achievable within typical *NAMD* simulations. For the examples in the subsequent sections, constant volume and temperature, *i.e.* *NVT*, ensembles were chosen in vacuum. Details of MD conditions are provided in the xMDFF scripts in the Supporting Material. Much of the analysis of the xMDFF-refined structures was performed with the *phenix.model_vs_data* package in *PHENIX*, including the computation of *R*_work_, *R*_free_ and *MolProbity* (Chen *et al.*, 2010 ▶) statistics. All analysis was performed on structures with *B* factors obtained through individual ADP refinement in *phenix.refine*.

#### 2.3.1. Refinement protocol   

xMDFF refinement is performed in multiple stages, tweaking the parameters as outlined here, which follows the general protocol employed for the cases presented in this paper. If the initial phasing model is thought to differ from the reference model by large-scale conformational changes (>∼2 Å r.m.s.d.), it is best to first only couple the backbone atoms to the density-derived potential. Furthermore, the global scaling factor ξ (3) is set to a low value, ∼0.1, which helps to reduce the overall force felt by each of the selected atoms owing to the density. Both of these settings allow the system to remain more flexible and not be heavily constrained to the map, which is likely to be quite noisy at the early stage of the refinement. The flexibility is also required for adequate sampling of the density map. As the r.m.s.d. of the system stabilizes with time, the side-chain atoms can be coupled to the density-derived potential and ξ can be increased to ∼0.3–0.5. Once the r.m.s.d. stabilizes again, it can be beneficial to further increase ξ and begin reducing the temperature of the system to 0 K. The refinement protocol is illustrated using the change of the r.m.s.d. of the phasing model relative to a known reference structure as shown in Supplementary Fig. S1 for a simple test system as described in §[Sec sec3.1]3.1.

As in other refinement techniques, xMDFF can also perform simulated annealing by increasing and subsequently decreasing the temperature of the system for multiple iterations to help avoid the structure becoming trapped in any local energy minimum. At this stage of the refinement it is important to frequently analyze the geometry, *R*_work_ and *R*_free_ of the structure. Poor geometries such as bad dihedral angles and a large difference between *R*_work_ and *R*_free_ can be indicative of overfitting a structure to the density and should be avoided.

Real-space refinement methods are expected to have a wide convergence radius, as has been formally shown, provided that the initial phases are of good quality (Diamond, 1971 ▶). molecular dynamics and simulated-annealing protocols further improved the convergence radius of real-space refinements (Brünger *et al.*, 1987 ▶). However, MD sampling of the side chains at low resolutions becomes computationally expensive. Historically, such issues have been addressed with dihedral sampling techniques (Rice & Brünger, 1994 ▶). As noted here, xMDFF uses simulated annealing to address the sampling of side chains. To increase the speed of fitting side chains and to improve their placement, future versions of xMDFF will incorporate data from rotamer libraries (Subramaniam & Senes, 2012 ▶). Improved fitting of side chains can also be achieved by using more realistic simulation environments. All of the refinements discussed here were performed in vacuum; however, it has been shown that MDFF-derived structures can be improved by the inclusion of explicit water molecules during the simulation, or with the use of a generalized Born implicit solvent for better computational performance with similar results (Tanner *et al.*, 2011 ▶). Additionally, membrane proteins can be simulated in a membrane, as in the previous case of MDFF studies of the ribosome (Frauenfeld *et al.*, 2011 ▶).

The computational cost of a typical xMDFF refinement will vary based on the system size and the number of iterations required, which is very system-dependent. Generally, we find that xMDFF can be computationally more demanding than other refinement software owing to the full molecular dynamics nature of the method. However, because xMDFF is an extension of MDFF, which was developed as part of *NAMD*, it is able to scale up from a single CPU core to thousands, potentially allowing the protocol to be applied to large systems such as the multi-million-atom HIV capsid (Zhao *et al.*, 2013 ▶). Furthermore, *NAMD*, and thus xMDFF, can utilize GPU acceleration to improve simulation performance (Stone *et al.*, 2007 ▶, 2010 ▶).

## 3. Results   

### 3.1. Proof of principle   

**Figure 3:**
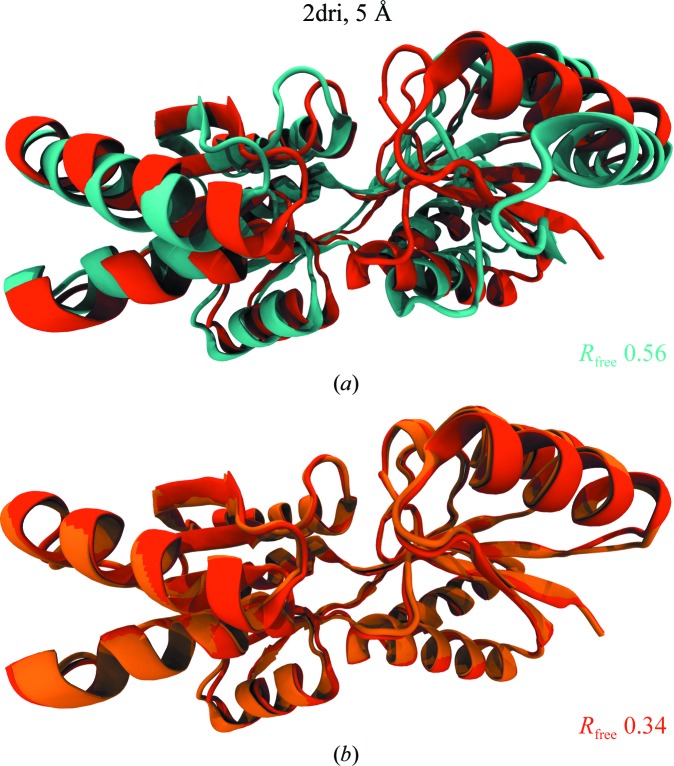
Test xMDFF refinement of the ‘closed’ conformation of d-ribose-binding protein using its ‘open’ conformation as the initial phasing model. Starting with an initial model of the open conformation (PDB entry 1urp; cyan) of the d-ribose-binding protein (*a*) and diffraction data from the target closed structure (PDB entry 2dri; orange) at resolutions ranging from 3.5 to 5 Å, xMDFF refinements were performed. (*b*) The xMDFF-refined closed structure (red) shows excellent agreement with the target at all resolutions (5 Å resolution shown with a final *R*_free_ of 0.34, down from an initial 0.56). Overall improvement was characterized using the *R*_free_ and cross-correlation of the final xMDFF structure and the r.m.s.d. of the final xMDFF structure to the target. Structural refinement is suggested by all three measures as well as the all-atom statistics provided in Table 1 ▶.

The performance of xMDFF was evaluated on a test structure, ribose-binding protein, with two known conformations at high resolution. The open conformation (PDB entry 1urp; 2.3 Å resolution) was used as an initial phasing model, with the closed conformation (PDB entry 2dri; 1.6 Å resolution) as a target model (Fig. 3 ▶). The refinement was performed using the diffraction data for the target model at four resolutions (3.5, 4, 4.5 and 5 Å), created by truncating the original intensities at each resolution limit. The four refinements began with the same initial phasing model and were evaluated against the same target; the final refined structures were evaluated using the overall improvement in *R*_free_ as well as the root-mean-squared deviation (r.m.s.d.) from the target model. xMDFF refinements improved the *R*_free_ value dramatically at every resolution, with an initial value of 0.57 and a final value of 0.23 at a resolution of 3.5 Å (Table 1 ▶). The all-heavy-atom r.m.s.d. for the refined structure at every resolution was 3.0 Å from the high-resolution 2dri target, down from the initial 5.46 Å. However, the final r.m.s.d. of the backbone alone at 3.5 Å resolution was 0.53 Å (down from an initial 4.46 Å), demonstrating proper backbone placement relative to the target model. In this case, the all-atom r.m.s.d.s are much higher relative to the backbone r.m.s.d.s because the target model was originally refined against 1.6 Å resolution data, where the side chains are much better resolved. Using the low-resolution synthetic data, the backbone can still be fitted quite well, but it is much harder to refine the side chains to the same extent.

To verify that xMDFF was performing as well as it could, a standard MDFF simulation was performed using density created directly from the 2dri target model and *F*_obs_ reduced to 3.5 Å resolution, which underwent non-iterative refinement. The final structure obtained from this fitting had an all-atom r.m.s.d. of 3.01 Å and a backbone r.m.s.d. of 0.56 Å, very close to those of the xMDFF refinements and demonstrating that xMDFF performs well against a more appropriate benchmark. Although the synthesized 3.5 Å resolution map manifests the best possible density at this resolution, it has much less side-chain information than the original 1.6 Å resolution map. This lack of side-chain density negatively affects side-chain fitting, and thus increased the side-chain r.m.s.d. relative to the known high-resolution target and accounts for the poor side-chain refinements in xMDFF. Since the initial model was originally obtained through refinement against high-resolution data, the overall structural geometry including the percentage of favored Ramachandran angles started very high at 98.5%. However, xMDFF did manage to improve the overall *MolProbity* (Chen *et al.*, 2010 ▶) scores, primarily through a decrease in rotamer outliers as well as in steric clashes. In practice, xMDFF improves the *MolProbity* statistics of almost all models discussed here owing to the MD-based nature of the method, which provides excellent structural restraints on the system.

We further tested the refinement capabilities of xMDFF by employing more realistic low-resolution synthetic data. After truncating the high-resolution data to 5 Å, the data were smoothed using a *B* factor of 35.00 Å^2^, in a fashion similar to the technique used in Schröder *et al.* (2010 ▶). Such a smoothing procedure further reduces the signal-to-noise ratio of the 5 Å resolution diffraction data, posing perhaps a more realistic refinement scenario. The refinement resulted in very similar but slightly worse results, with a backbone r.m.s.d. of 0.93 Å, an *R*_free_ of 0.34 and an *R*_work_ of 0.28. To test the robustness of xMDFF refinements to the choice of initial structure, the 5 Å resolution diffraction data were refined using another search model that has the same overall r.m.s.d. from the target but with a shift in the positioning of the helices. The new search model, shown in Supplementary Fig. S1, has a backbone r.m.s.d. of 4.45 Å from 2dri, which is comparable to that of 1urp, but it also has an r.m.s.d. of 1.5 Å relative to 1urp. The refinement results are slightly worse but very similar, with an r.m.s.d. of 0.8 Å, a final *R*_free_ of 0.34 (down from an initial 0.62) and a final *R*_work_ of 0.26 (down from 0.56).

Additionally, during refinements of the test model the scaling factor which determines the overall strength of the steering forces being applied was varied in order to determine the optimal parameterization for future work (see §[Sec sec2]2 for additional information). A lower scaling factor was determined to be useful during early stages of backbone refinement to keep the system flexible, but increasing the scaling factor is considered useful as refinement progresses in order to couple the structure more strongly to the density and improve the fit; however overfitting needs to be avoided at this stage.

### 3.2. Improving refinements of reported PDB structures   

To test whether xMDFF is capable of improving previously refined structures, it was applied to six structures at 4–4.5 Å resolution deposited previously into the PDB (Fig. 1 ▶). The structures served as an initial phasing model and xMDFF was able, without any further knowledge of the reference model, to improve the *R*_free_ by at least 0.01 (PDB entry 1aos) and up to a maximum of 0.08 (PDB entries 1av1 and 1xdv) in the case of all six structures (Table 1 ▶). Furthermore, the *R*_free_ and *R*_work_ values of each system were relatively close, indicating that xMDFF is not overfitting the structures. Additionally, every xMDFF-refined structure exhibits an improved structural geometry, as shown by a higher percentage of Ramachandran favored angles and a lower overall *MolProbity* score over those of the initial structures (Table 1 ▶). In one of the most improved cases (PDB entry 1xdv), the main cause for the lowered *R*_free_ was improvement in a highly flexible region with relatively large root-mean-square fluctuations (Supplementary Fig. S2).

**Figure 4:**
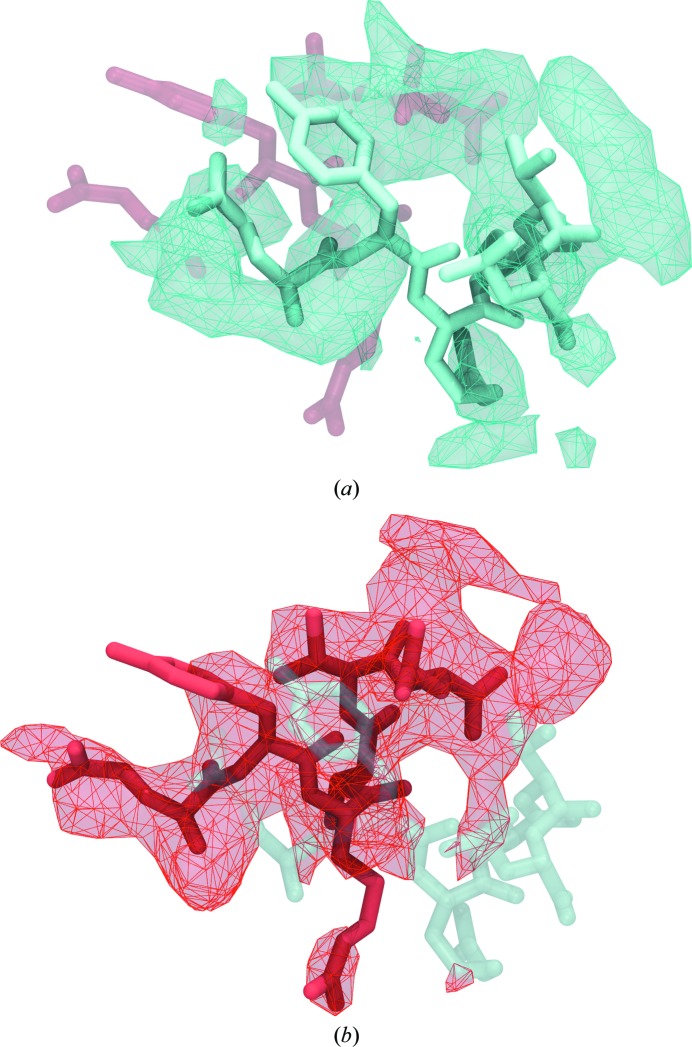
Refinement of the highly flexible region in the case of PDB entry 1xdv. Substantial density improvements are observed in a flexible region of 1xdv, illustrated by the difference in the density map between the initial (blue) (*a*) and xMDFF-refined final (red) (*b*) structures; local cross-correlations increase from 0.47 to 0.63, implying a more unambiguous placement of the atoms.

This region shifted the most during xMDFF refinement, with an r.m.s.d. of 4.3 Å from the initial to the final model (part of which is shown in Fig. 4 ▶). Flexible regions often diffract poorly and can be difficult to properly place using traditional means of X-ray refinement, especially at low resolution. Densities were generated using *F*_obs_ and also ϕ_calc_ from the initial and final models, respectively (Fig. 4 ▶). A significant improvement in the quality of the densities can be seen in terms of their respective completeness and how well the structure fits inside the density, with the latter captured by an increase in the local CC from 0.47 (initial, blue) to 0.63 (final, red).

The application of force-field-based MD simulations for model refinement inherently provides geometries consistent with some energy minima. Not only does the procedure improve protein conformations, but it also conserves the structures of cofactors, keeping them compatible with the surrounding protein. For example, xMDFF refinement of human hemoglobin (PDB entry 1ye1) preserves the planar coformation of the heme group. Consequently, distortions in cofactors accompanying multiple conformational states of protein crystals can be accounted for by low-resolution diffraction data (Supplementary Fig. S3*a*).

xMDFF provides the atomic positions as well as the chemical bonding in the low-resolution X-ray map. In many cases, the assumed connectivity between atoms is reflected in the degree of structural refinement, which in turn can affect predictions of the associated function. For example, in the case of the pentameric cobratoxin (Cbtx)-bound acetylcholine-binding protein (AChBP) complex (PDB entry 1yi5), the complex has a dense core composed primarily of the AChBPs. The cobratoxins are composed of two antiparallel β-sheets forming slightly concave discs, five of which emerge from the dense AChBP core, with each disc sporting ten cysteine residues. However, given only the positions of atoms it is unclear whether the cysteines are in oxidized or reduced states, *i.e.* whether disulfide bridges are present or not. Using the 4.2 Å resolution diffraction data, xMDFF refinements were performed assuming either the presence or the absence of the disulfide bridges (Supplementary Fig. S3*b*). A more pronounced refinement was achieved with the assumption of oxidized cysteines (a final *R*_work_ of 0.26 and an *R*_free_ of 0.29 in contrast to the final *R*_work_ of 0.27 and *R*_free_ of 0.31 for the reduced state), implying the existence of disulfide bridges. The presence of the five xMDFF-predicted disulfide bridges per Cbtx disc has been validated in biochemical studies, whereby four bridges are key to the stability and the concave shape of the disc and the fifth is resposible for optimal Cbtx–AChBP binding (Bourne *et al.*, 2005 ▶). Thus, xMDFF provided biomolecular structures with energetically favorable geometries that interpret low-resolution density maps as well as help to clarify structures’ relevant biological functions.

The xMDFF-based structures [PDB entries 1av1 (Borhani *et al.*, 1997 ▶), 1xdv (Sondermann *et al.*, 2004 ▶), 1yi5 (Bourne *et al.*, 2005 ▶), 1aos (Turner *et al.*, 1997 ▶), 1jl4 (Wang *et al.*, 2001 ▶) and 1ye1 (Kavanaugh *et al.*, 2005 ▶)] in Fig. 1 ▶ showed improvement with regard to *R* factor and geometry over structures that had resulted from conventional approaches, *e.g.* *REFMAC* or *CNS*/*X-PLOR* (PDB entries 1yi5, 1jl4, 1ye1, 1av1, 1xdv and 1aos), applied to the same diffraction data, most likely owing to the combination of starting xMDFF with good initial phases from an already refined search model and having molecular dynamics incorporated in xMDFF. An identical conclusion on the use of good initial phases was drawn from *X-PLOR* refinements with MD simulations. However, models 1jl4 and 1av1, which were published in 2001 and 1997, respectively, resulted from early-generation refinement methods and accordingly had relatively high *R*-factor values, posing an easy challenge for our present xMDFF treatment. In other cases (1yi5 and 1ye1) the original refinements involved initial *REFMAC* or *X-PLOR* rigid-body fitting of the search model used and the authors had to resort to manual fitting employing *FRODO* to account for the needed structural flexibility. The automated, force-field-based flexible fitting algorithm in xMDFF should avoid errors arising in manual fitting. Altogether, for the discussed examples, the xMDFF-refined models are found to be better or at least as good as the published models.

### 3.3. xMDFF refinement and experimental validation of the Ci-VSP crystal structure   

**Figure 5:**
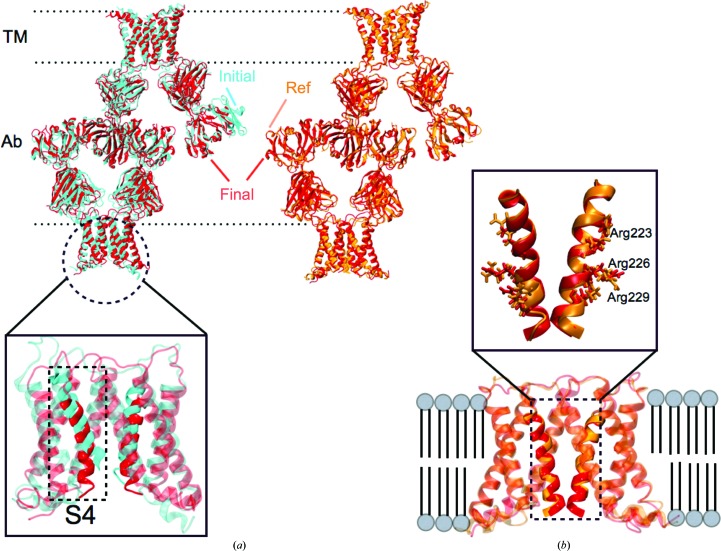
xMDFF refinement of the voltage-sensing protein Ci-VSP. (*a*) A *MUFOLD*-predicted homology model (cyan) was used as an initial phasing model in xMDFF; this model has an r.m.s.d. of 6 Å from an independently refined Ci-VSP structure (orange; Li *et al.*, 2014 ▶). Ci-VSP includes a transmembrane (TM) domain and was crystallized with an antibody (Ab). Inset: the placement of the S4 helix within the TM region which determines the voltage-gating capabilities of the protein is of particular interest. (*b*) xMDFF refinement with 4 Å resolution diffraction data produced a final structure (red) 2.6 Å away from the independently refined Ci-VSP structure (orange). Inset: the placement of the S4 helix in the Down position and the alignment of the regularly placed voltage-sensing Arg residues is in agreement with the independently refined model (Li *et al.*, 2014 ▶).

Finally, xMDFF was applied to solve the structure of a voltage-sensing protein, Ci-VSP, using 3.6, 4 and 7 Å resolution diffraction data (Fig. 5 ▶). To validate the reliability of the xMDFF refinement, the work was carried out in parallel with an independent experimental investigation of the structure and function of Ci-VSP (Li *et al.*, 2014 ▶).

Voltage-sensing protein is a common scaffold present in voltage-gated ion channels, voltage-sensitive enzymes and voltage-gated proton channels, which are related to diverse important physiological functions. As illustrated in Fig. 5 ▶, the protein under current investigation, Ci-VSP, is arranged as an antiparallel four-transmembrane-helix bundle S1–S4; the overall structure is in agreement with the basic three-dimensional architecture of all known voltage-sensor proteins (Jiang *et al.*, 2003 ▶; Long *et al.*, 2007 ▶; Payandeh *et al.*, 2011 ▶; Zhang *et al.*, 2012 ▶). The positively charged S4 helix within Ci-VSP reorients upon stimulus from a transmembrane electric field, leading to downstream responses. Despite a wealth of structural and functional data, the details of this conformational change remain controversial, in particular the movement of the S4 helix.

According to electrophysiological results, Ci-VSP at 0 mV assumes the resting (Down) state in the wild type (WT) but the activated (Up) state in the R217E mutant. Crystal structures of both states have been determined experimentally: R217E at 2.5 Å resolution and WT at 3.6 Å resolution (Li *et al.*, 2014 ▶). Unfortunately, there was crystallographic uncertainty in the S4 position in the Ci-VSP WT 3.6 Å resolution electron-density map. Spectroscopic data had limited the S4 position of Ci-VSP WT to three options in reference to the R217E structure: no conformational change, one click down and two clicks down, where a click refers to the offset of a helix by one turn. Obviously, the confirmation of the S4 position in Ci-VSP WT became the key to the puzzle. xMDFF was applied to predict the WT Ci-VSP structure and, thereby, to resolve the uncertainty in the low-resolution data.

Refinement started from a *MUFOLD*-predicted (Zhang *et al.*, 2010 ▶) medium-confidence homology model developed using information from 13 proteins (Supporting Information). During refinement, the tentative model underwent a remarkable large-scale deformation with an r.m.s.d. of 5.96 Å. Unlike many traditional refinement techniques, xMDFF is able to handle such large-scale structural deformations between the initial and final structures, producing in the present case final *R*_free_ values of 0.28 and 0.29, starting from initial *R*_free_ values of 0.50 and 0.48, at 3.6 and 4 Å resolution, respectively (Table 1 ▶). Positioning of the functionally relevant S4 helices is in excellent agreement with the one-click-down model of WT Ci-VSP, with an r.m.s.d. ranging from 0.4 to 1 Å.

To further confirm the S4 position, potential structural models were generated by gradual shift and rotation of the S4 helix from the Up-conformation model to the two-click-down model in 2000 even steps (in all a ∼10 Å vertical displacement and ∼110° rotation). Two independent parameters for model evaluation were calculated from each of these 2000 structures: (i) the crystallographic *R*_free_ value and (ii) the correlation coefficient (CC) between the experimental density map of the S4 region and the calculated electron density from the refined model (Supplementary Fig. S4). Fig. 5 ▶(*b*) shows that the structure corresponding to the *R*_free_ minimum and CC maximum from Supplementary Fig. S4 resides in a region that unambiguously places the position of the S4 helix in the one-click-down position. Assuming that movement of the S4 helix in Ci-VSP follows the classic helix-screw or sliding-helix mode (Li *et al.*, 2014 ▶), other degrees of freedom that are not involved in any helix-screw type of motion are considered to be redundant. Subsequently, the *R*_free_ minimum identified by xMDFF along the chosen line of helix-screw offsets is likely to be global for the resting state of WT Ci-VSP. xMDFF clearly differentiates the pattern of side chains associated with a specific S4 helix position even though the individual side chains are not fully visible at 3.6 Å resolution. The resulting S4 position is three residues lower than that of the Up structure, positioning Arg residues into the protein interior and away from lipids, and is in excellent agreement with the one-click-down model.

The refinement with the 7 Å resolution data was not as pronounced as those with the 3.6 and 4 Å resolution data. However, improvements were still observed in the r.m.s.d. and *R* factors. The *R* factors from the resulting structure were considerably higher than those obtained from higher resolutions. Unlike the higher resolution maps, the 7 Å resolution data failed to distinguish between the Up and one-click-down models of Ci-VSP. Furthermore, using the 7 Å resolution data the *R* factors derived from the high-resolution Up or one-click-down structures were comparable to that of the xMDFF output. Thus, we conclude that the 7 Å resolution map is too coarse to resolve any information relevant to S4 placement.

The present example reaffirms the capability of xMDFF to address large-scale structural deformations, as has been shown for the test case with synthetic data sets, to produce significant refinements yielding realistic structures, but now from more noisy low-resolution experimental data.

## 4. Discussion   

The introduction of MD and simulated-annealing algorithms have facilitated structure determination from low-resolution diffraction data sets (Brünger *et al.*, 1987 ▶). xMDFF provides a significant step forward among the MD-based algorithms as a crystallographic refinement tool. A brief comparison of xMDFF predictions with those from other available refinement methods is provided in Supplementary Table S2. Broadly speaking, xMDFF refinements provide the lowest *R* factors, minimal overfitting and improved structural statistics among the methods compared [DEN in both *CNS* and* PHENIX* (Brünger *et al.*, 1998 ▶; Schröder *et al.*, 2010 ▶) in addition to default *PHENIX* (Adams *et al.*, 2010 ▶) refinement]. However, as further discussed in the Supporting Information, although we try to achieve a fair comparison, the results might depend on the user’s knowledge of the system, the application of the software and the usage of manual fitting. For a more controlled comparison, xMDFF was used to refine two structures using the same initial models (PDB entries 3kso and 3k0i) and reflection data (PDB entry 3k07) from a previous comparison of *Rosetta*–*PHENIX* (DiMaio *et al.*, 2013 ▶), *CNS* DEN (Brünger *et al.*, 1998 ▶; Schröder *et al.*, 2010 ▶), *PHENIX* (Adams *et al.*, 2010 ▶) and *REFMAC*5 (Murshudov *et al.*, 2011 ▶). The two structures are sufficiently low resolution, >∼4 Å, and the search models are displaced by >∼5 Å relative to the target. In the case of 3kso, xMDFF achieved a better refined structure than the best one from DiMaio and coworkers, which was obtained using *CNS* DEN (using *R*_work_ and *R*_free_ as the determinants). This improvement is evident in the lower *R*_work_ (0.2518), *R*_free_ (0.3509) and *MolProbity* score (4.03) than those obtained for the *CNS* DEN-refined structure (*R*_work_ 0.319, *R*_free_ 0.387, *MolProbity* score 4.15). When starting with the 3k0i search model, the xMDFF-refined structure has a higher *R*_work_ (0.3226) and *R*_free_ (0.3944) but a lower *MolProbity* score (3.00) than those obtained for the best refined structure, again using *CNS* DEN (*R*_work_ 0.307, *R*_free_ 0.368, *MolProbity* score 3.94). However, xMDFF still produces better results than the rest of the methods in the study. It should also be noted that while the *R*_free_ and *R*_work_ of *Rosetta*–*PHENIX*-refined structures in these two cases are worse than those with *CNS* DEN, they have much better *MolProbity* scores of 2.00 for 3ks0 and 1.91 for 3k0i, a trend that is observed in all but two of the test cases.

Additional considerations must be made to satisfactorily, if not conclusively, compare the multiple refinement protocols. Restricting discussions to only the real-space refinement protocols compared here brings forth several differences. For example, elastic networks, as implemented in DEN-based protocols, robustly predict collective global motions but do not work as well for describing local changes. Therefore, additional care should be taken when dealing with side chains through the use of library-based refinement protocols. Furthermore, elastic network or heurestic force-field-based algorithms often require optimization of the interatomic interactions with each new class of applications. Universal force-field-based protocols such as ours are expected to be more physically correct as the atomic interactions are calibrated against very accurate quantum calculations. However, sometimes the application of different force fields leads to different results owing to differences in the calibration protocol used for force-field development. Thus, any further comparison of the various real-space refinement protocols is beyond the scope of this paper.

xMDFF guides the dynamics of a search model to a refined structure through use of first principles *via* universal force fields together with restraints from the X-ray maps. Consequently, manual real-space fitting, as is commonly used with reciprocal-space refinement protocols, is often avoided. This benefit is of relevance to protocols in drug discovery, which often require determination of multiple crystal structures distributed over a broad range of conformations or ligand-bound states. If performed manually, refinement in drug discovery requires extensive repetition of the same task for each structure. xMDFF naturally provides a semi-automated computational platform to systematically perform several model-building and repetitive refinement steps amenable to high-throughput crystallography.

The initial xMDFF implementation presented here has several limitations that we hope to address through future development. Firstly, the quality of the refinement results might depend on the nature of the force field used, the effects of which require further investigation. Additionally, the present implementation is unable to handle changes in secondary structure during the simulation, and thus the refinement is strongly biased by the folds present in the search model. Any change in the secondary structure can only be invoked at the homology-modelling stage and not during the refinement. Finally, the current xMDFF implementation is limited to refinement of biomolecules for which force fields are already available. Refinement of nonbiological systems is subject to force-field availibility.

In summary, the application of xMDFF to synthetic as well as experimental low-resolution X-ray data demonstrated the capability of the software to refine phasing models 6 Å away from target data even with maps as coarse as 7 Å resolution, a feat thus far achieved, to the best of our knowledge, very rarely in low-resolution X-ray crystallography. MD naturally provides the necessary sampling required to flexibly fit a model into an electron-density map. Through flexible fitting into iteratively updated model-phased maps, xMDFF can bring about a series of large-scale deformations of the initial structure relevant to its refinement. Detailed features characterizing macromolecular function, such as the location of helices in voltage-sensor proteins or disulfide-bridge networks in cobratoxin, have been accurately determined. The application of force fields within xMDFF achieves realistic atomic structures with sterically and conformationally acceptable geometries. Low *MolProbity* scores together with a consistently small difference between *R*_work_ and *R*_free_ values imply negligible overfitting in xMDFF refinements. The quality of the overall refinement is confirmed *via* improvements in cross-correlations with simulated density. Finally, xMDFF output structures require little post-processing to initiate MD simulations for subsequent analysis of their dynamics in a host medium. In summary, xMDFF together with sequence information and homology modelling provides a general approach to determining all-atom structures from low-resolution X-ray data. 

## Supplementary Material

Supplementary Figures and Tables.. DOI: 10.1107/S1399004714013856/rr5069sup1.pdf

Information, configuration files, and scripts for running xMDFF software.. DOI: 10.1107/S1399004714013856/rr5069sup2.gz

## Figures and Tables

**Table 1:** Table summarizing the quality of xMDFF-refined structures The closed conformation of ribose-binding protein (PDB entry 2dri) was refined against four synthetic diffraction data sets with resolution 3.5–5 Å using an open conformation (PDB entry 1urp) of the same protein as the initial model. Six previously reported low-resolution (4–4.5 Å) structures from the Protein Data Bank showed further refinement with xMDFF. Finally, the structure of a voltage-sensor protein under investigation (Li *et al.*, 2014 ▶) was determined using xMDFF refinement of an initial homology model. For further quality evaluations, please see Supplementary Table S1.

		Ramachandran favored (%)		*MolProbity* score		*R*_free_		*R*_work_		R.m.s.d. (Å)	
PDB code	Resolution (Å)	Initial	Final	Initial	Final	Initial	Final	Initial	Final	Initial	Final
2dri	3.5	98.51	97.01	1.85	0.77	0.62	0.30	0.58	0.23	5.46	3.01
										4.46†	0.53†
	4.0	98.51	96.64	1.85	0.72	0.54	0.30	0.54	0.22	5.46	3.02
										4.46†	0.55†
	4.5	98.51	95.90	1.85	0.92	0.57	0.37	0.52	0.24	5.46	3.06
										4.46†	0.68†
	5.0	98.51	96.27	1.85	0.85	0.56	0.34	0.53	0.23	5.46	3.05
										4.46†	0.67†
1av1	4.0	90.20	95.01	3.70	1.94	0.42	0.34	0.38	0.33	N/A	
1xdv	4.1	95.30	95.05	2.87	2.01	0.41	0.33	0.39	0.29		
1yi5	4.2	86.99	90.72	3.08	1.73	0.31	0.29	0.26	0.26		
1aos	4.2	89.14	92.17	3.40	2.45	0.24	0.23	0.20	0.21		
1jl4	4.3	87.15	91.03	3.24	1.47	0.42	0.38	0.35	0.33		
1ye1	4.5	77.25	84.72	2.68	1.89	0.27	0.24	0.26	0.26		
4g80 (Ci-VSP)	3.6	89.20	92.96	2.86	2.10	0.50	0.28	0.49	0.26	5.96	2.47
										5.75†	1.84†
	4.0	89.20	92.14	2.86	2.10	0.48	0.29	0.48	0.27	5.96	2.60
										5.75†	2.06†
	7.0	89.20	90.73	2.86	2.18	0.46	0.40	0.45	0.38	5.96	3.36
										5.75†	2.78†

† Backbone-only r.m.s.d.

## Footnotes

1 Supporting information has been deposited in the IUCr electronic archive (Reference: RR5069).
