# Supplementary material for: xMDFF: molecular dynamics flexible fitting of low-resolution X-ray structures
Source: Acta Crystallogr D Biol Crystallogr. 2014 Aug 29;70(Pt 9):2344–55. doi: 10.1107/S1399004714013856 (PMC4157446; doi:10.1107/S1399004714013856)
Supplement: Supplementary file 1 [file d-70-02344-sup1.pdf]

Ryan McGreevy, Abhishek Singharoy, Qufei Li, Jingfen Zhang, Eduardo Perozo, Klaus Schulten

xMDFF: Molecular Dynamics Flexible Fitting of Low Resolution X-Ray Structures

Email: kschulte@ks.uiuc.edu
Tel: 1-217-244-1604

Supplementary Information:

- Complete table of refinements
- Figures
- Homology information for CiVSP search model
- Comparison of xMDFF refinements to those from PHENIX and PHENIX.DEN

ID	Resolution (Å)	Ramachandran Favored %		Molprobity Score		R-free		R-work		RMSD (Å)	
		initial	final	initial	final	initial	final	initial	final	Initial	final
2DRI	3.5	98.51	97.01	1.85	0.77	0.62	0.30	0.58	0.23	5.46<br>4.46*	3.01<br>0.53*
	4.0	98.51	96.64	1.85	0.72	0.54	0.30	0.54	0.22	5.46<br>4.46	3.02<br>0.55*
	4.5	98.51	95.90	1.85	0.92	0.57	0.37	0.52	0.24	5.46<br>4.46	3.06<br>0.68*
	5.0	98.51	96.27	1.85	0.85	0.56	0.34	0.53	0.23	5.46<br>4.46	3.05<br>0.67*
1AV1	4.0	90.20	95.01	3.70	1.94	0.42	0.34	0.38	0.33	N/A	
1XDV	4.1	95.05	95.30	2.87	2.01	0.41	0.33	0.39	0.29		
1YI5	4.2	86.99	90.72	3.08	1.73	0.31	0.29	0.26	0.26		
1AOS	4.2	89.14	92.17	3.40	2.45	0.24	0.23	0.20	0.21		
1JL4	4.3	87.15	91.03	3.24	1.47	0.42	0.38	0.35	0.33		
1YE1	4.5	77.25	84.72	2.68	1.89	0.27	0.24	0.26	0.26		
Ci-VSP	3.6	89.20	92.96	2.86	2.10	0.50	0.28	0.49	0.26	5.96<br>5.75*	2.47<br>1.84*
	4.0	89.20	92.14	2.86	2.10	0.48	0.29	0.48	0.27	5.96<br>5.75	2.60<br>2.06*
	7.0	89.20	90.73	2.86	2.18	0.46	0.40	0.45	0.38	5.96<br>5.75	3.36<br>2.78*

ID	Ramachandran allowed %		Ramachandran outlier %		Rotamer outliers		Cbeta deviation		Clash score	
	initial	final	initial	final	initial	final	initial	final	Initial	final
2DRI	1.12	2.61	0.37	0.37	17	2	0	1	3.51	0.25
	1.12	2.61	0.37	0.75	17	2	0	1	3.51	0.00
	1.12	2.99	0.37	1.12	17	0	0	0	3.51	0.25
	1.12	2.99	0.37	0.75	17	0	0	0	3.51	0.25
1AV1	3.35	4.46	0.17	0.53	90	6	0	0	40.11	5.22
1XDV	4.16	3.93	0.81	0.57	78	21	2	1	30.58	9.48
1YI5	9.89	7.38	3.92	1.91	192	12	4	0	8.66	4.09
1AOS	7.36	5.84	3.50	1.99	128	32	1	1	23.62	3.46
1JL4	10.41	6.92	2.01	2.41	49	6	1	3	30.42	0.92
1YE1	21.10	13.88	2.76	1.38	39	1	14	2	17.48	10.50
Ci-VSP	3.78	6.02	7.02	1.02	82	31	134	0	5.00	3.18
	3.78	5.35	7.02	2.51	82	47	134	10	5.00	4.88
	3.78	4.37	7.02	4.90	82	58	134	10	5.00	4.18

Table S1 Improvements in R-factors, structural statistics as summarized by the Ramachandran distribution, MolProbity and clash scores, and RMSDs from the target structure resulting from xMDFF refinements of eight low-resolution (4-4.5Å) X-ray structures. * implies backbone only RMSDs.

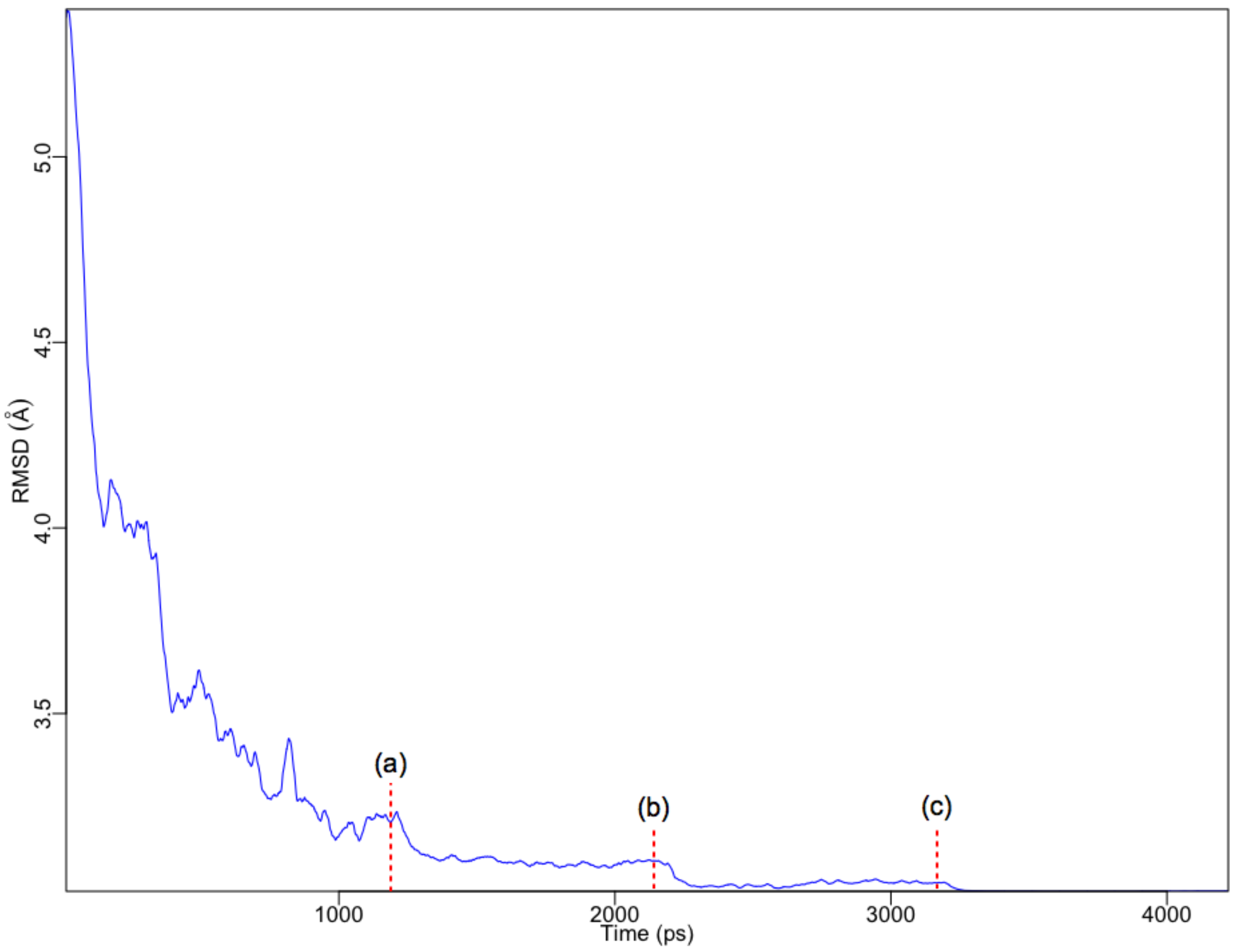

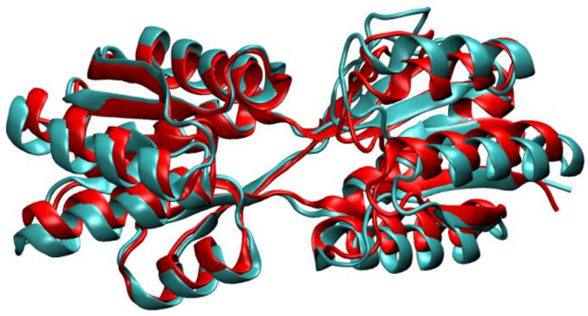

(d)

Figure S1 Test xMDFF refinement of the “closed” conformation of D ribose binding protein using its “open” conformation as initial phasing model at four different resolutions. An overall decrease in the root mean square deviation (RMSD) of the phasing model relative to its known “closed” target implies refinement of the former during the xMDFF simulation. The protocol is labeled on the plot for the refinement at 4 Å referring to steps indicating an initial coupling of C-alpha atoms to the map with a global scaling factor, ξ, of 0.1 **(a)**, followed by coupling of backbone to the map with a

global scaling factor, ξ, of 0.3 **(b)**, coupling the backbone and side-chains with a ξ of 0.6 **(c)**, and finally reducing the temperature from 300 K to 0 K. Beta factor sharpening also began at step (b). Fluctuations about the mean decreasing RMSD trend are representative of the map noise, global scaling factor and thermal regime at various states of the refinement and, thus, are reduced as the temperature and noise decreases, and global scaling factor increases. **(d)** Alternative search model for the refinement of the closed conformation of D ribose binding protein, showed in cyan, which is 1.5 Å RMSD relative to 1URP (red) and 4.45 Å away from the known target, 2DRI. Overall, the new search model is laterally expanded relative to 1URP.

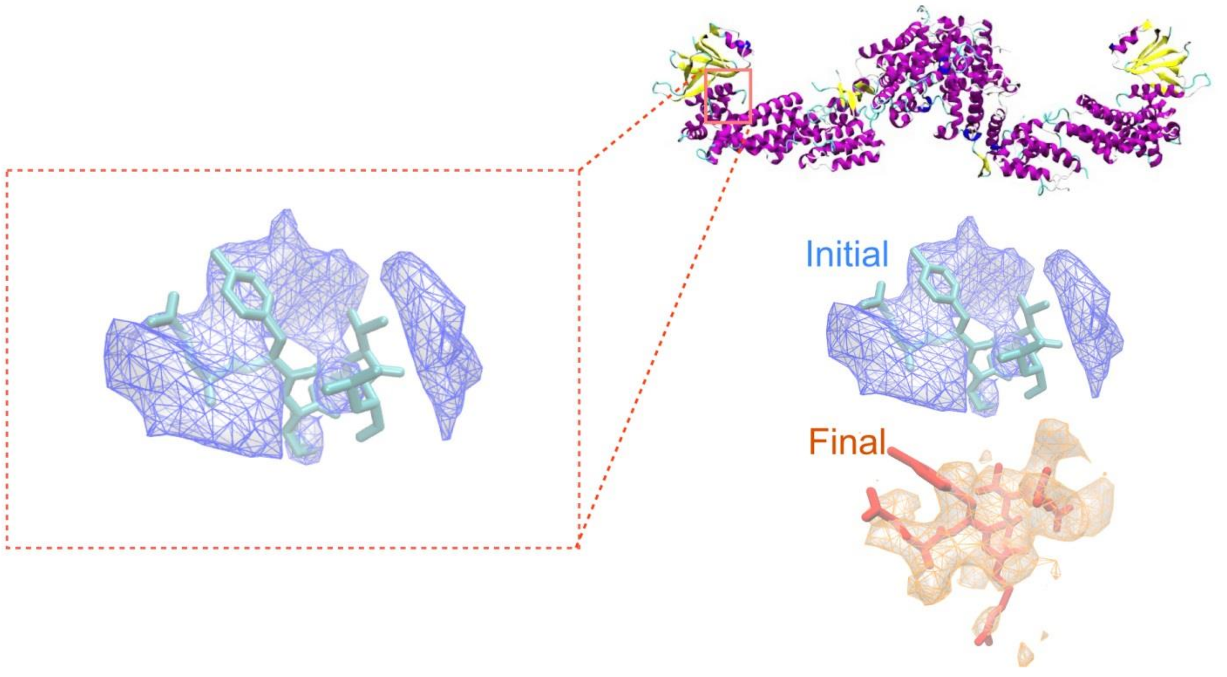

Figure S2 xMDFF Refinement of 1XDV zooming in on computations involving the most flexible loop regions located at the apical end of the protein.

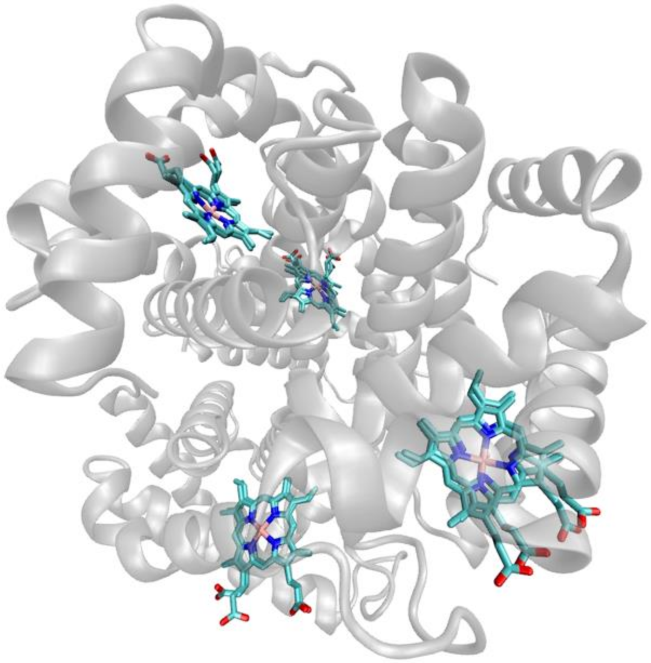

(a)

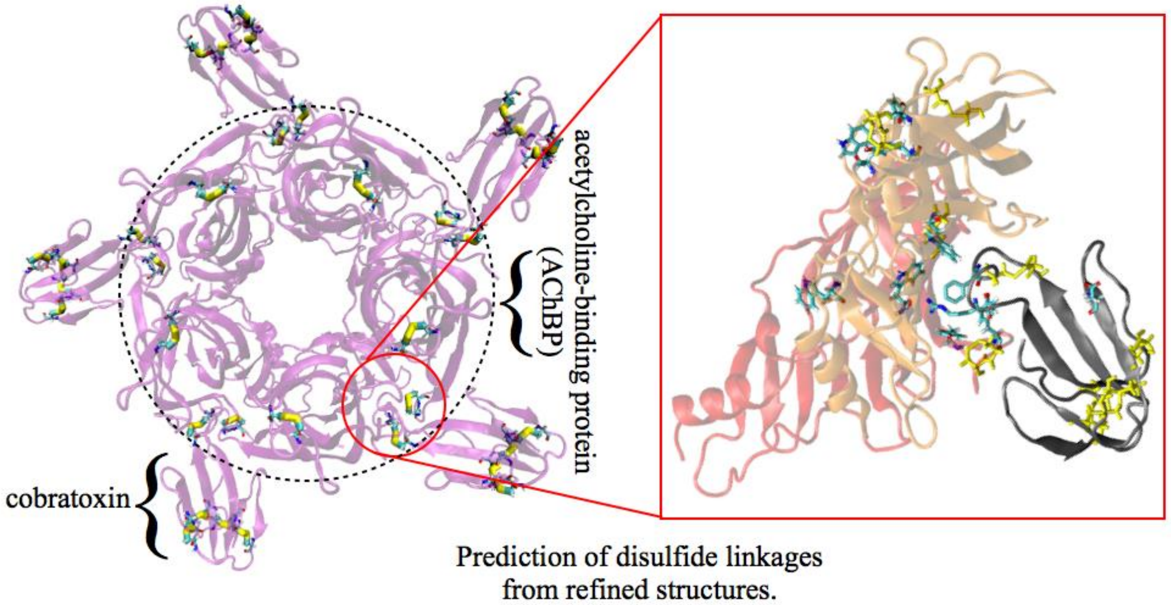

(b)

Figure S3 **(a)** Hemoglobin structure showing planarity of the heme groups are conserved during xMDFF refinement. **(b)** Structure of the cobratoxin (Cbtx) bound acetylcholine-binding protein (AChBP) complex demonstrating key disulfide bonds (yellow) for maintaining the Cbtx shape and AChBP-Cbtx stability.

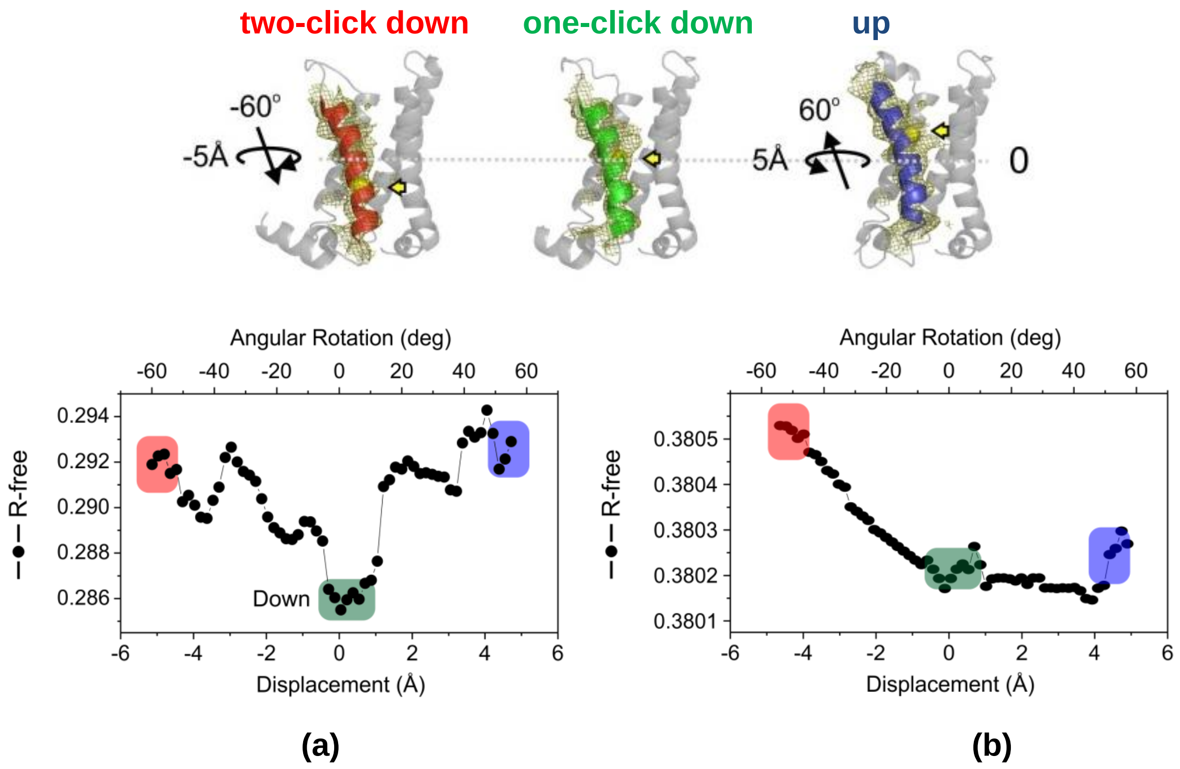

Figure S4 Top: Three potential models within the electron density map of Ci-VSD WT: Two-click down (S4 in red), One-click down (S4 in green) and Up-conformation (S4 in blue). The rotation angle and vertical displacement of S4 were measured in reference to the One-click down model. R-free values are plotted using the **(a)** 3.6 and **(b)** 7 Å diffraction data against angular and vertical displacement of the S4 helix from its xMDFF-determined down position. At 3.6 Å the three helix conformations are sufficiently distinguishable suggesting the down orientation provides the lowest R-free value, and, therefore, the best fit. In contrast, the 7 Å data fail to distinguish the down and up conformations.

Template used for MUFOLD predicted search model:

Structures of Ci-VSP are modeled employing chains from the following thirteen homologous proteins.

Sequence:
QFRVRAVIDHLGMRVFGVFLIFLDIILMIIDLSLPGKSESSQSFYDGMALALSCYFMLDLG
LRIFAYGPKNFFTNPWEVADGLIIVVTFVVTIFYTVLDEYVQETGADGLGRLVVLARLLR
VVRLARI

PDB ID Chain
4DXW C
3RW0 B
4EKW D
3RVZ B

Sequence:
EVQLVESGGGLVQPGGSLRLSCAASGFNVSSSSIHWVRQAPGKGLEWVASISPSSGY
TSYADSVKGRFTISADTSKNTAYLQMNSLRAEDTAVYYCARKQYSYWRDAAWAMDY
WGQGTLVTVSSASTKGPSVFPLAPSSKSTSGGTAALGCLVKDYFPEPVTVSWNSGAL
TSGVHTFPAVLQSSGLYSLSSVVTVPSSSLGTQTYICNVNHKPSNTK
VDKKVEP

PDB ID Chain
3EFF B
2QQK H
3KYM B
3KR3 H

Sequence:
DIQMTQSPSSLSASVGDRVTITCRASQSVSSAVAWYQQKPGKAPKLLIYSASSLYSGV
PSRFSGSGSGTDFTLTISSLQPEDFATYYCQQHQYNSLIFGQGTKVEIKRTVAAPSVFIF
PPSDEQLKSGTASVVCLLNNFYPREAKVQWKVDNALQAGNSQESVTEQDSKDSTYSL
SSTLTLSKADYEKHKVYACEVTHQGLSSPVTKSFNR

PDB ID Chain
1FE8 L
3IVK L
2FGW L
3UC0 L
3BN9 C

Comparison of xMDFF refinements:

ID	Resolution	Molprobity					R-free					R-work				
		initial	final				initial	final				initial	final			
	(Å)		xMDFF	Phenix	PD	CD		xMDFF	Phenix	PD	CD		xMDFF	Phenix	PD	CD
2DRI	3.5	1.85	0.77	2.82	3.07	4.36	0.62	0.30	0.59	0.56	0.59	0.58	0.23	0.52	0.54	0.42
	4.0	1.85	0.72	3.69	5.08	4.47	0.54	0.30	0.51	0.48	0.59	0.54	0.22	0.39	0.29	0.45
	4.5	1.85	0.92	3.76	4.78	4.29	0.57	0.37	0.55	0.51	0.55	0.52	0.24	0.41	0.50	0.37
	5.0	1.85	0.85	2.83	4.52	4.13	0.56	0.34	0.54	0.46	0.54	0.53	0.23	0.50	0.28	0.46
Ci-VSP	3.6	2.86	2.10	3.62	4.83	3.87	0.50	0.28	0.45	0.41	0.39	0.49	0.26	0.48	0.34	0.33
	4.0	2.86	2.10	3.11	4.60	3.90	0.48	0.29	0.43	0.45	0.41	0.48	0.27	0.48	0.37	0.35
	7.0	2.86	2.18	3.62	4.81	N/A	0.46	0.40	0.42	0.43	N/A	0.45	0.38	0.31	0.33	N/A

Table S2 Comparison between xMDFF, PHENIX, PHENIX.DEN (PD) and CNS.DEN (CD) refinements of the D ribose binding and voltage sensor proteins via evaluation of the Molprobity scores and R-factors of the refined structures.

The quality and accuracy of xMDFF predicted models are evaluated via comparison with structures from PHENIX, PHENIX.DEN, CNS.DEN, and REFMAC5 refinements. For a given system, e.g., 2DRI or Ci-VSP, all refinements started with the same search model. Results presented in Table S2 strongly suggest that xMDFF refinements provide the lowest R-factors, minimal overfitting, and improved structural statistics. In comparison, traditional PHENIX refinements result in much less improvement of the search model. Larger scale deformations are evident in the DEN refinements, and PHENIX.DEN even provides lower R-factors than PHENIX. However, DEN refinements have been found to induce more overfitting than xMDFF. The overfitting is reflected in the greater difference between the associated R_{work} and R_{free} as well as high Molprobity scores of the DEN refined structures. REFMAC5 results are similar to those from PHENIX and are therefore not presented.

The differences between xMDFF, PHENIX, DEN and REFMAC5 results are, to a certain extent, expected. xMDFF being an MD-based real-space refinement method requires minimal human intervention during refinement. In xMDFF, principles of molecular physics as reflected in the universal force-fields together with restraints from the X-ray maps guide the dynamics of the search model to a refined structure. In contrast, traditional methods such as PHENIX or REFMAC often require a combination of computational inverse-space and manual real-space refinement tools to address large scale deformations, and subsequently get to very high quality refinements. To fairly evaluate the advantages of using xMDFF, one needs to compare only the computational part of other refinement protocols to that of xMDFF. Since manual fittings were not allowed in any of the refinements of Table S2, results from only the inverse space protocol are relatively poor.

DEN results are more promising in terms of addressing large scale deformations necessary to resolve low resolution crystal structures. However, the default DEN protocol as implemented in PHENIX, used for the present refinements, suffers from overfitting issues. In fact, DEN has been successfully used in the past for 2DRI

refinement [1]. But, unlike employing truncated experimental data in Table S2, less noisy entirely synthetic data had been used to test DEN refinement. Thus, the older DEN results should not be compared to the present answers. Also, CNS.DEN could not resolve the 7 Å data for CiVSP.

One should also note that much better results could be obtained by an investigator familiar with the structure and flexibility of parameters in the refinement protocol. Nevertheless, in our endeavor to provide as unbiased a comparison as possible, the refinements have been performed with experimental collaborators (co-authors) adept in the use of REFMAC5 and PHENIX, and core members from the DEN development group.

The PHENIX refinement protocol included rigid_body + individual_sites + individual_adp + tls refinements with default secondary structure and hydrogen bonding restraints, simulated annealing for each of the 10 macrocyles. For DEN refinement with Phenix, the default parameters were used in addition to secondary structure restraints; for CNS however, such restraints are not applicable.

[1] Schroder, G. F., Brunger, A. & Levitt, M. Combining efficient conformational sampling with a deformable elastic network model facilitates structure refinement at low resolution. *Structure* **15**, 1-12 (2007).
